# The anti-neoplastic effect of doxycycline in osteosarcoma as a metalloproteinase (MMP) inhibitor: a systematic review

**DOI:** 10.1186/s13569-020-00128-6

**Published:** 2020-04-30

**Authors:** Argyris C. Hadjimichael, Athanasios F. Foukas, Olga D. Savvidou, Andreas F. Mavrogenis, Amanda K. Psyrri, Panayiotis J. Papagelopoulos

**Affiliations:** 1 3rd Department of Orthopedic Surgery, KAT Hospital, Athens, Greece; 2 1st Department of Orthopedic Surgery, National and Kapodistrian University of Athens, Medical School, Attikon University hospital, Athens, Greece; 3 Department of Internal Medicine, Section of Medical Oncology, National and Kapodistrian University of Athens, Medical School, Attikon University hospital, Athens, Greece

**Keywords:** Osteosarcoma, Metalloproteinase, VEGF, Doxycycline, Metastasis

## Abstract

**Background:**

Osteosarcoma is a very aggressive primary bone tumour, affecting mainly young populations. Most cases diagnosed have distant macro- and micro-metastases at the time of diagnosis. Surgical resection with neoadjuvant and adjuvant therapies improves the overall and disease-free survival of patients. Doxycycline, a synthetic tetracycline, has been found to act either as an antibiotic drug or as a chemotherapeutic agent. Its anti-neoplastic role has been found to be significant, in vitro and in vivo laboratory trials, in various types of cancer, such as prostate, intestinal, central neural system cancers and osteosarcoma. Inhibition of metalloproteinases (MMPs) in different stages of tumour expansion is the most well-understood mechanism. MMPs are secreted molecules from various normal cells, such as fibroblasts, leucocytes and vascular smooth muscles, as well as from cells with high proliferative potential, such as tumour cells. In osteosarcoma, MMPs have been found to be overexpressed. MMPs help osteosarcoma cells survive, grow and produce metastases in distant sites, mainly in the lungs. Doxycycline blocks extracellular matrix and basic membrane degradation by suppressing MMP function. As a consequence, osteosarcoma cells lose their ability to invade and metastasize. Additionally, doxycycline eliminates the secretion of vascular endothelial growth factor (VEGF) and deprives the supply of circulating nutrients by its anti-angiogenesis action. The aim of this review is to evaluate doxycycline’s action against osteosarcoma cells as an MMP-inhibitor and interpret its usage as a chemotherapeutic agent.

**Methods:**

We checked PubMed and Google Scholar for recently published data, on the tumour-supportive role of MMPs and VEGF in osteosarcoma cells. We further studied published experimental trials on the role of doxycycline as a tumour-suppressive agent via MMPs and VEGF inhibition.

**Results:**

MMPs and VEGF have been found to play a fundamental role in osteosarcoma cells survival and high aggressiveness by in vitro, in vivo and clinical trials. Nevertheless, doxycycline has proved its tumour-suppressive effect by in vivo experimental trials in various cancers but not yet in osteosarcoma.

**Conclusion:**

Doxycycline remains a promising chemotherapeutic agent against osteosarcoma via MMP inhibition, showing the need for further in vivo and clinical trials to be carried out in the future.

## Background

Osteosarcoma, also known as osteogenic sarcoma, is the most common primary bone tumour with highly metastatic and lethal behaviour [1]. In the United States of America, approximately 800–900 new cases of osteosarcoma are diagnosed every year [1]. The age distribution of osteosarcoma is bimodal, as the first age peak is recorded between the first and third decades and the second smaller peak (10% of cases) in the sixth decade [1]. Osteosarcoma accounts for 2% of all cancers in childhood and remains a challenging disease to prevent and treat [1]. For localized and resectable osteosarcomas, the 5-year survival rate varies from 60 to 80%, but for metastatic lung disease, the survival rate diminishes to 40% [1]. The lung is the most common site for initial metastasis, as approximately 10% of osteosarcoma patients have pulmonary nodules at the time of diagnosis [2].

Proliferation of tumour cells, increasing tumour size and local invasion combined with new vessels formation for necessary nutrients and oxygen intake are basic steps for osteosarcoma cells to expand and produce distant metastases [3]. Osteosarcoma progression is strictly associated with extracellular matrix (ECM)-degrading matrix metalloproteinases (MMPs), which play a fundamental role in cancer survival and invasion, as well as with the development of a neoplastic vascular network [3]. MMPs are zinc-dependent endopeptidases secreted by fibroblasts, leucocytes, vascular smooth cells and rapidly proliferating tumour cells [4]. The biological attributes of MMPs appear in various physiological and pathological processes, such as collagen and elastin degradation, endothelial cell formation during angiogenesis, migration of vascular smooth muscles and proliferation along with migration of tumour cells [4]. Currently, in humans, there are 23 well-identified MMPs that stimulate cancer survival and expansion, which represent a target group for anti-cancer drugs [5].

**Fig. 1:**
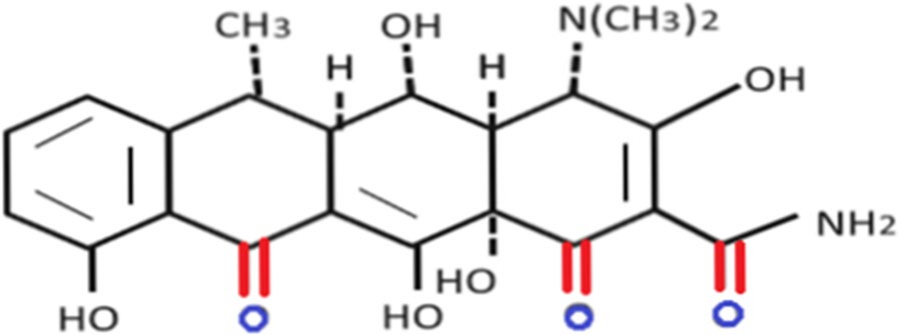
The chemical structure of Doxycycline. The lower oxygen-rich part is able to form chelation bonds with MMPs [7]

Doxycycline, a chemically modified tetracycline, is an inexpensive drug with a safe profile. It is the only MMP inhibitor that acts in a concentration-dependent manner, approved by the US Food and Drug Administration for the treatment of periodontal disease [6]. It is composed of a four-ring core with an attached dimethyl amino group at the C4 carbon on the upper site of the molecule. At the lower oxygen-rich part of doxycycline, the formation of chelation bonds with Zn^2+^ and Ca^2+^ ions within MMPs can occur [7]. Despite its anti-microbial usage, recent observations have shown that doxycycline has a cytotoxic effect on tumour cells. Inhibition of MMPs secreted by osteosarcoma cells can reduce neo-angiogenesis and tumor invasiveness by suppressing ECM destruction and preventing the formation of micro-metastasis at the very early stages of cancer expansion. As a prophylactic chemotherapeutic agent, doxycycline could improve the overall survival rate in patients suffering from osteosarcoma (Fig. 1).

## Method

### Search strategy

We searched for published articles from PubMed and Google Scholar. Our first aim was to mention pre-clinical osteosarcoma trials to link the important role of several subtypes of MMPs with the level of invasiveness and metastatic attitude of osteosarcoma cells. Second, we checked bibliographies for the effect of MMPs and VEGF in clinical trials of osteosarcoma to guarantee that their neoplastic behaviour has been proven not only in experimental models but also in real osteosarcoma human specimens. After we recognized the tumourigenic action of MMPs, we focused our search on the anti-neoplastic effect of doxycycline in various types of cancer to ensure that it acts not only as an antibiotic drug but also as a tumor-suppressive agent via MMP inhibition. Additionally, we searched for the indirect anti-neoplastic effect of doxycycline by its VEGF-suppressing action that has been observed in malignant cells. Finally, we studied all published pre-clinical trials interpreting the anti-neoplastic effect of doxycycline in osteosarcoma experimental models to identify its action on osteosarcoma cells.

### Selection criteria

Three inclusion criteria were applied:

1. Recent published articles. We mainly focused on literature from 2013 to 2019.
2. Published articles evaluating the metastatic effect of MMPs in osteosarcoma pre-clinical trials.
3. Data from in vivo experimental trials, investigating the anti-tumour effect of doxycycline as an MMP inhibitor in various types of human cancer.

Exclusion criteria:

1. Published in vitro and in vivo research on the anti-MMP effect of other tetracyclines on osteosarcoma cells.
2. Research on MMP’s activity in preclinical trials of other types of human cancer.
3. Experimental trials evaluating the usage of doxycycline in non-human cancer cells or syngeneic animal models.

## Results

Thirty-seven out of 49 (75.5%) cited articles were first published between 2013 and 2019. From the 1999 to 2013 interval, we observed a lack of experimental trials and published data referring to the antineoplastic effect of doxycycline in osteosarcoma.

### Studies’ content

We found only three in vitro pre-clinical trials, interpreting the anti-metastatic behaviour of doxycycline and no in vivo experimental trials on osteosarcoma animal models that examined the blockage of metastases in distant organ from 1997 to 2020.

### The metastatic role of MMPs in osteosarcoma in pre-clinical trials

MMP-1 (also known as collagenase-1) has been found to be markedly upregulated after intra-tibial injection of 143B highly metastatic human osteosarcoma cells into SCID mice compared with injection of a non-metastatic HOS cell line [8]. MMP-1 plays an important role in the lung formation of micro- and macro-metastases in patients diagnosed with osteosarcoma [8]. The expression of MMP-2 (also known as gelatinase-A) promotes invasion and metastases of osteosarcoma cells. After engraftment of MNNG/HOS cells in NOD/SCID/IL2rγnull mice, the stabilizing protein N-α-Acetyltransferase prevented degradation of MMP-2 and increased the ability of tumor cells to metastasize [9]. Inhibition of microRNA-328 by resveratrol (3,5,4′-trihydroxystilbene, a natural polyphenol) suppressed MMP-2, and the ability of MNNG/HOS osteosarcoma cells to metastasize after intra-tibial injection in SCID mice [10]. The expression of MMP-2 is elevated in patients with osteosarcoma, especially in those with pulmonary metastases, and could be an independent prognostic marker for the total survival time after initial diagnosis of the primary tumour [11]. Additionally, circulating MMP-2 seems to alter sensitiveness of osteosarcoma cells in chemotherapeutic drugs as a shift from MMP-9 to MMP-2 secretion is correlated with poor response to them [12]. The malignant phenotype in osteosarcoma cells is obviously promoted by another metalloproteinase, the MMP-3 (also known as stromelysin-1) [13]. The migration and invasion properties of MMP-3 knockdown, MG-63 and TE85 highly metastatic osteosarcoma cells were extraordinarily deteriorated [13]. Osteosarcoma cells overexpress the very important metalloproteinase, MMP-9 (also known as gelatinase-B) that helps them to invade and metastasize. The hairy/enhancer-of-split related with YRPW motif protein 1 (HEY1) has been found to upregulate the expression of MMP-9 in nude mice injected with HEY1 shRNA-transfected 143B cells, increasing their ability to migrate [14]. Additionally, MMP-9 has been found to be involved in osteosarcoma cell invasion, with a highly predictive role in the development of lung metastases, especially when co-expressed with chemokine CXCR4 [15–17]. Inhibition of MMP-11 (also known as stromelysin-3) in MG‑63 and U2OS osteosarcoma cells by upregulating the micro RNA-125a-5p has proved the important function of another MMP during the migration and invasion process [18]. MMP-13 (also known as collagenase-3) secreted by osteosarcoma cells has been found to be another important molecule for the ability of tumour cells to invade the extracellular matrix and induce lung metastases [19, 20]. The downregulation of MMP-13 by injected MicroRNA-143 in highly metastatic human osteosarcoma cells, 143B, significantly suppresses lung metastases, but the proliferation potential remains the same [19]. Conversely, upregulation of MMP-13 and stimulation of the AKT-pathway mediated by MMP-13 by Interleukin-32 enhances the motility and invasion of osteosarcoma cells, confirming the key role of this metalloproteinase in lung metastases [5, 20] (Table 1).

**Table 1:** This table shows the degradation of collagenous and non-collagenous substrates by important MMPs

Metalloproteinase (MMP)	MMP-substrates	Osteosarcoma experimental trial	Metastatic action
MMP-1 (collagenase-1)	Collagen (I, II, III, VII, VIII, X), casein, entactin, laminin, pro-MMP-1, -2, -9, and serpin	Intratibial injection of 143B cells with shRNA-downregulated MMP-1 expression in SCID mice [8]	↓lung micro- and macro-metastases than control-group
MMP-2 (gelatinase A)	Gelatin, collagen (IV–VI, X), elastin, fibronectin	1. MNNG/HOS cells in NOD/SCID/IL2rγnull mice treated with N-α-Acetyltransferase [9]	↑ MMP-2 ↑metastases
		2. Intratibial injection of MNNG/HOS cells in SCID mice treated with resveratrol [10]	↓ microRNA-328 ↓MMP-2 ↓ metastases
MMP-3 (stromelysin-1)	Laminin, aggrecan, gelatin, fibronectin	MMP-3 knockdown in MG-63 and TE85 osteosarcoma cells [13]	↓MMP-3 ↓metastases
MMP-9 (gelatinase B)	Gelatin, collagens (IV, V, VII, X, XIV), elastin, fibrillin, osteonectin	Control or HEY1 shRNA-transfected 143B cells inoculated in knee joint of nude mice [14]	↑HEY1 ↑MMP-9 ↑metastases
MMP-11 (stromelysin-3)	Fibronectin, laminin, aggrecan, gelatin	Human osteosarcoma cell lines (HOS, Saos-2, MG-63 and U2OS) with downregulation of miR‑125a‑5p [18]	↓miR‑125a‑5p ↑MMP-11 ↑metastases
MMP-13 (collagenase-3)	Collagen (II, III, IV, IX, X), proteoglycans, fibronectin, laminin, elastin	1.Athymic nude mice inoculated with 143B cells in knee treated with or without miR‐143(targets plasminogen activator inhibitor-1) [19]	↑ miR‐143 ↓MMP-13 ↓metastases
		2. Knockdown of IL-32 in MG-63 osteosarcoma cells [20]	↓IL-32 ↓MMP-13 ↓invasiveness

In vitro and in vivo osteosarcoma trials have indicated the key role of these MMPs in tumor metastasis [21]

### The prognostic value of MMPs and VEGF expression in clinical trials

The detection of osteosarcoma using MMP-9 as a biomarker has been found to be extremely accurate in a metanalysis of Wang et al. in which a total of 892 patients in different clinical stages were included [22]. Foukas et al. attempted in 2002 to interpret the prognostic metastatic value of ΜMP-9 in 55 patients with stage IIB osteosarcoma around the knee. Immunohistochemical studies in resection specimens of these patients showed that overexpression of MMP-9 in osteosarcoma cells is significantly associated with metastases and poor overall survival [23]. However, the prognostic value of serum MMP-9 levels for metastases in patients suffering from osteosarcoma was found to be statistically non-significant [23]. A study by Han et al. among 177 cases diagnosed with osteosarcoma revealed a correlation between high levels of serum alkaline phosphatase (ALP) and MMP-9 for the prediction of metastatic disease and poor prognosis [24]. On the other hand, a metanalysis by Wen et al. among five cohort studies revealed that the expression of MMP-2 has a strong value for the prognosis of metastases and increased mortality of patients suffering from osteosarcoma [25].

Elevated levels of various MMPs can increase the expression of vascular endothelial growth factor (VEGF) and vice versa [4]. VEGF is the most important molecule for establishing a neoplastic network of new blood vessels, and it conserves the ability of malignant cells to survive and spread to distant organs. Liu et al. found among 84 osteosarcoma samples that high expression of VEGF is significantly correlated with a high possibility of metastases and poor prognosis in patients suffering from osteosarcoma [26]. A systematic meta-analysis by Chen et al., in 2013, among 12 studies with a total of 559 patients suffering from osteosarcoma, revealed that VEGF expression is obviously associated with lower overall survival due to a higher incidence of metastases [27].

**Fig. 2:**
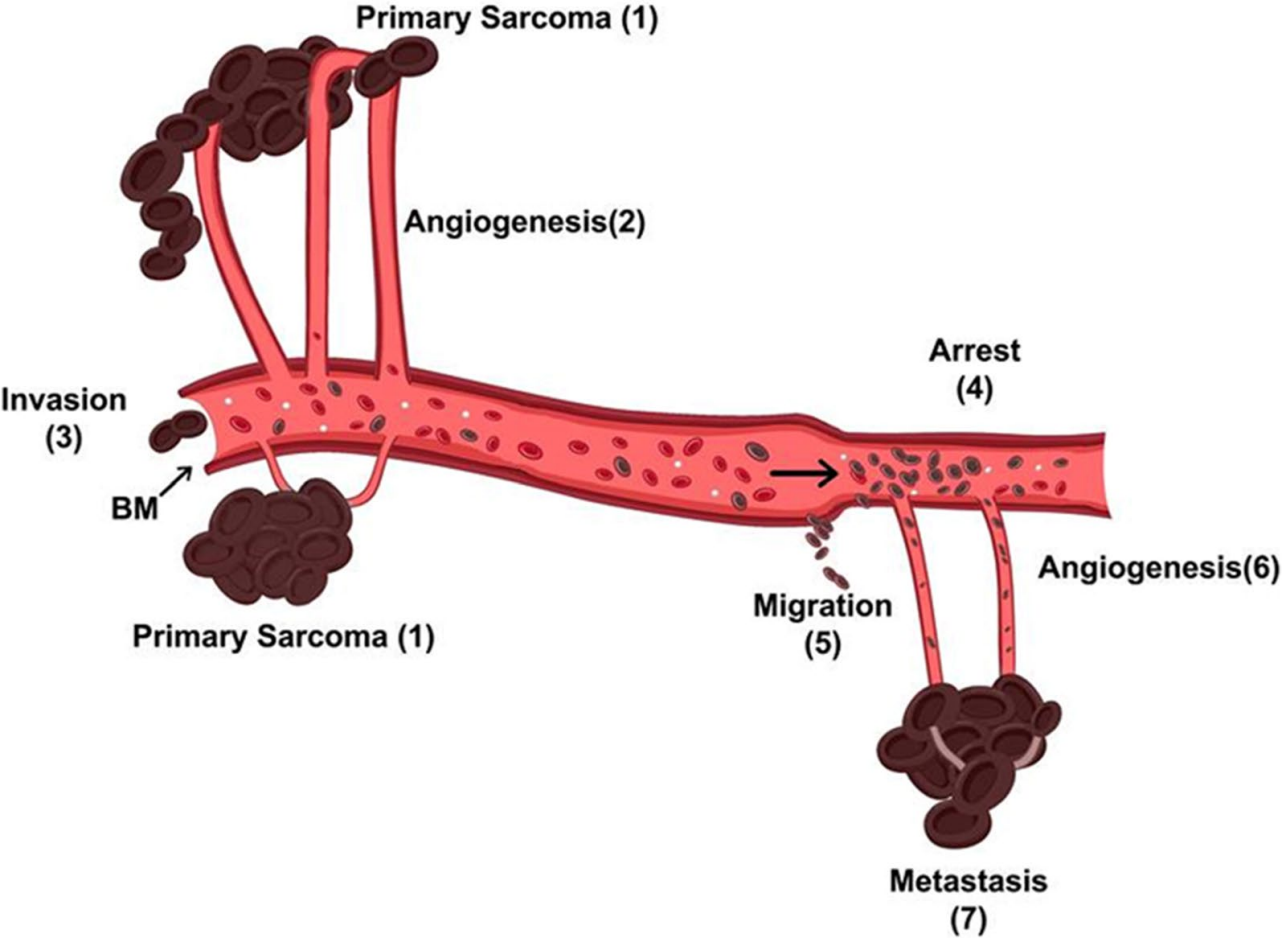
The role of neo-angiogenesis for tumor metastasis from local to distant sites. (1) The local proliferation and expansion of primary osteosarcoma cells. (2) MMPs cause overexpression of VEGF leading to new blood vessels formation. (3) Degradation of extracellular matrix and basic membranes by MMPs help malignant cells to invade in blood vessels. (4) Osteosarcoma cells arrested from endothelial cells of blood vessels in distant organs. (5) Tumor cells migrate in distant sites following blood circulation where they adhere. (6) Secondary tumors secrete MMPs in order to form a sufficient neoplastic blood vessels network. (7) Osteosarcoma cells in distant tissues create mature metastatic tumors [4]

The strict correlation between osteosarcoma lung metastases and poor survival, along with MMPs and VEGF overexpression, motivated research on inhibiting agents. One of them is probably doxycycline (Fig. 2).

### The anti-neoplastic role of doxycycline as an MMP inhibitor in various human cancer cells

Doxycycline, as an MMP inhibitor, mediates extracellular matrix degradation and acts as an anti-proliferative, anti-invasive and anti-angiogenic agent, deteriorating the expanding and invasive potential of tumour cells. The molecular mechanism that supports MMP inhibition by doxycycline involves the following different assumptions: (i) MMP-mRNA becomes unstable, (ii) inactivation of MMPs via Zn^2+^ chelates, (iii) elimination of reactive oxygen species secreted by ECM cells that activate pro-MMPs, (iv) blockage of MMP activation via the MT1-MMP pathway [28]. Inactivation of MMPs secreted by tumour cells deprives the formation of new capillary vessels, which could support the survival of malignant cells and provide a potential frame for invasion [29]. As a consequence, doxycycline acts as an indirect cytotoxic drug and prohibits the formation of pulmonary micro- metastases. Tumor cells that have already settled in the lungs require the formation of a supportive angiogenic environment to grow into clinically detectable metastases. The anti-metastatic action of doxycycline has been proven in different types of cancer.

A doxycycline-treated group and a control group of engrafted human MHCC97H cells of hepatocellular carcinoma into BALB/c mice showed that doxycycline acted as an MMP-2 and MMP-9 inhibitor [30]. Doxycycline suppressed the growth of tumour cells and prolonged mouse survival. The mechanism of vasculogenic mimicry, in which the formation of mosaic vessels from endothelium and tumour vessels along with bridging channels in tumour cells has been found to be occluded via doxycycline administration, with malignant cells losing the potential to migrate in distant organs [30]. In this study, doxycycline inhibited the viability, proliferation, migration and invasion of hepatocellular carcinoma cells in vitro [30]. However, the authors missed to examine in vivo the presence or absence of distant metastases (e.g. lungs, other organs) between the doxycycline-treated and non-treated xenograft groups. We believe that this lack of evidence consists a limitation for this study.

The suppressive effect of doxycycline against oral squamous cell carcinoma has been associated with MMP inhibition [31]. The overproduction of MMPs in this type of cancer leads to lymph node and distant organ metastases. The administration of doxycycline can decrease tumour invasiveness in an in vitro SCC-15 cell line and tumour growth volume in vivo in xenografted nude mice [31]. After 24 h treatment of SCC-15 cells, MMP-9 mRNA levels were found to be significantly reduced, while MMP-2 secretion deteriorated in the post-transcriptional stages via Zn^2+^ chelation with doxycycline. It is obvious, to our knowledge, that doxycycline can eliminate MMPs either at the pre- transcriptional or post-transcriptional level acting as an adjuvant anti-invasive chemotherapeutic drug [31]. Shen et al. used a dose of 3 mg/mice/day for their doxycycline-treated xenograft model [31]. Doxycycline exerted a significant suppressive effect on tumor growth for this in vivo osteosarcoma model.

The inhibitory effects of doxycycline on the expression of MMPs in prostate cancer are another example of its anti-metastatic usage. NF-kB signalling has been found to regulate the expression of MMPs in the nuclei of lipopolysaccharide (LPS)-induced PC3 human prostate cancer cells [32, 33]. Doxycycline treatment downregulated the levels of MMP-2, MMP-8, MMP-9 and NF-kB in a dose-dependent manner [33], which represents another important molecular pattern that restricts tumour cell proliferation, invasion and angiogenesis.

The cytotoxic effect of doxycycline via MMP inhibition was also demonstrated with in vitro and in vivo pre-clinical trials of xenografted HuTu-80 human duodenal adenocarcinoma cells in mice [33]. A daily intraperitoneal dose of 40 mg/kg doxycycline was administered to the duodenal adenocarcinoma murine model of this trial [33].

The anti-neoplastic effect of doxycycline on intestinal neoplasias has already been confirmed previously by Sagar et al. in human colorectal cancer cells (HT29 cell line) [34]. Doxycycline has been found to work on its own as an apoptotic inducer with anti-proliferating and anti-invasive action against HT29 cells [34]. The synergistic action of doxycycline with cisplatin and oxaliplatin, the most commonly used platinum compounds for the cancer chemotherapy was not confirmed in this study [34].

A study by Onoda et al. revealed that doxycycline causes dose-dependent inhibition of cell-growth on human colorectal cancer cell lines, LS174T and HT29 [35]. MMP-2 and MMP-9 expression were not inhibited by doxycycline at a dose of 5 or 10 μg/dl, but they were down-regulated by this concentrations [35].

**Fig. 3:**
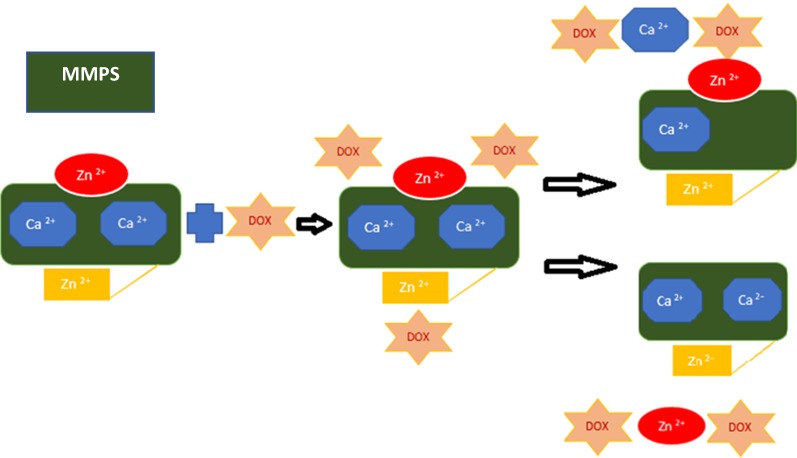
Mechanism of MMPs inactivation by Doxycycline. Osteosarcoma cells secrete MMPs which support survival and invasiveness of malignant cells. Doxycycline (DOX) inactivates MMPs by forming chelation bonds with their Zn^2+^ and Ca^2+^ ions [7]

Glioblastoma multiforme is the most common brain cancer found to be susceptible to doxycycline, after in vitro treatment of highly aggressive U251HF human cells [36]. Doxycycline decreased extracellular levels of MMP-2 after the formation of chelation bonds, but it did not downregulate MMP-2 mRNA at pre-translational stages [36]. As a consequence, an anti-proliferative effect and reduced cell invasiveness occur due to MMP inhibition by doxycycline (Fig. 3).

### Anti-angiogenetic effect of doxycycline via MMP-inhibition

MMPs provoke angiogenesis, which is crucial for tumour viability, cells proliferation and metastasis in distant organs. MMPs contribute to angiogenesis in two distinct ways. First, MMPs degrade the basement membrane and extracellular matrix and provide the opportunity for endothelial cells of existing vessels to migrate and settle in new positions, creating a new supporting capillary network for tumour cells [21]. Second, the degradation of extracellular matrix releases angiogenic factors such as vasculo-endothelial growth factor (VEGF), fibroblast growth factor (bFGF) and tumour growth factor (TGFβ), which trigger intracellular angiogenic pathways for new vessels formation [21, 29]. VEGF also increases vessel permeability and allows tumour cells to enter blood circulation and move to distant sites. Additionally, these angiogenetic factors induce secretion of MMPs by endothelial cells, conserving high amounts of local MMPs in the tumour area [21]. In vitro inhibition of VEGF expression in U2OS osteosarcoma human cells promoted tumour cells apoptosis and reduced cell proliferation through vascular insufficiency [37]. Chen et al., in a meta-analysis of 12 studies, showed that secretion of VEGF is a powerful prognostic factor for the total survival rate of patients suffering from osteosarcoma. Among 559 patients, overall disease-free survival was strictly associated with the expression levels of VEGF [27].

**Fig. 4:**
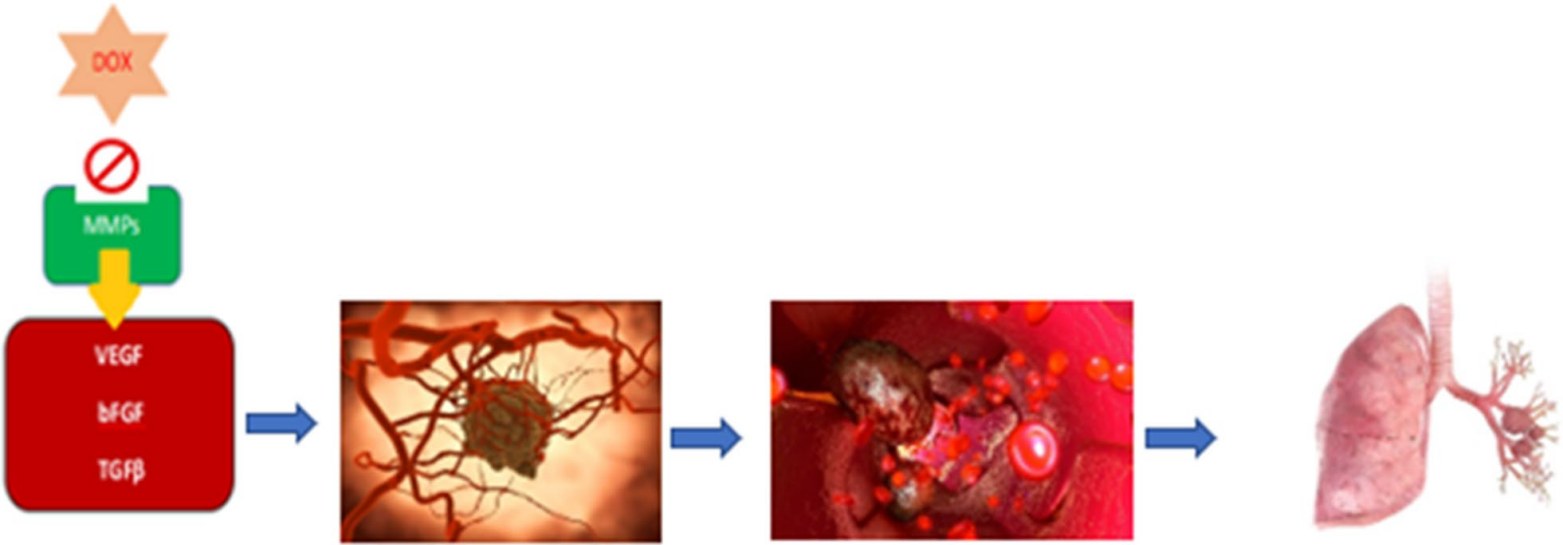
The indirect anti-angiogenesis effect of Doxycycline via MMPs inhibition. MMPs increase the secretion of proangiogenic factors like VEGF, bFGF and TGF promoting the formation of new network of blood vessels. Adequate blood supply helps osteosarcoma cells to survive, proliferate and penetrate blood vessels to give metastases in distant organs, mainly in lungs. Doxycycline acts as a cytotoxic agent by inhibiting MMPs and depriving tumor cells from nutritional necessities [35]

According to Merentie et al. doxycycline reduced the expression of VEGF in endothelial cells in vitro in a dose-dependent manner, but failed to downregulate its expression in transgenic mice in vivo [38]. In contrast, doxycycline has been found to eliminate angiogenesis indirectly as an MMP-9 inhibitor via suppression of VEGF expression in cerebral matrix of a mouse model [39]. Additionally, doxycycline proved to increase tissue inhibitors of metalloproteinases-1 (TIMP-1) and stop human aortic smooth muscle cell (HASMCs) migration by inhibiting VEGF expression [40]. As an MMP inhibitor doxycycline represents a promising anti-angiogenetic agent due to its anti-VEGF action (Fig. 4).

### Inhibition of MMPs by doxycycline in pre-clinical osteosarcoma models: in vitro studies

Fife et al. were the first in 1997 to study the anti-proliferative and apoptotic effects of doxycycline in cultured human osteosarcoma cells in vitro [41]. Their hypothesis that doxycycline, a non-toxic antibiotic drug could, act either as an MMP-2 inhibitor or as an anti-neoplastic chemotherapeutic agent in osteosarcoma cells proved realistic. Osteosarcoma cells from six patients and one human osteosarcoma cell line, U2OS, were cultured in the presence or absence of 5 mg/ml and 10 mg/ml doxycycline. Their pre-clinical trial showed that doxycycline at a dose of 10 mg/ml suppressed cell proliferation by three- to seven-fold in osteosarcoma cultures. MMP-2 activity was significantly inhibited only at a dose of 5 mg/ml [41].

Cakir and Hahn, in 1999 described the suppressive effect of Doxycycline on MMP-1, beyond its antimicrobial usage. Their in vitro study on canine osteosarcoma cells, showed that 5 mg/ml and 10 mg/ml of Doxycycline, could minimize tumor volume 50% and 75%, respectively [42]. MMP-1 secretion, reduced 35% and 50% at Doxycycline’s doses of 10 mg/ml and 20 mg/ml, respectively [42]. Their in vitro trial, proved the antiproliferative usage of a chemically modified tetracycline, against osteosarcoma cells via MMP-1 inhibition [42].

The most recent study on the inhibitory effect of doxycycline in the pediatric human osteosarcoma cell line U2OS was conducted by Roomi et al. in 2013 [43]. Osteosarcoma cells proved to be sensitive to doxycycline exposure, as MMP-2 and MMP-9 secretion were suppressed in a dose-dependent manner. Gelatinase zymography revealed total blockage of MMP-2 at 5 mg/ml and blockage of MMP-9 at 10 mg/ml doxycycline [43]. As MMPs were recognized in several experimental trials as crucial molecules for the invasive properties of tumour cells, doxycycline was interpreted as a potential adjuvant and neoadjuvant chemotherapeutic drug.

### The estimated human equivalent dose of doxycycline correlated with minimal toxicity

Doxycycline has been tested in different doses to evaluate its anti-proliferative and tumor-suppressive action. We found a wide range of administered doses between several studies. For example, Shen et al. used a doxycycline dose of 3 mg/mice/day for SCC-15 xenografted nude mice [31], while Galván‑Salazar et al. used a doxycycline dose of 40 mg/kg for xenografted HuTu-80 nude mice [33]. We believe that the minimum anti-metastatic circulating levels of doxycycline has not been sufficiently evaluated as the lung and distant organs were not examined for metastases with tissue biopsy. According to Lucchetti et al. a single intraperitoneal administration of 10 or 100 mg/kg in 7-week-old male C57BL76 mice reach a peak-plasma concentration from 2 to 10 μg/ml. This concentration is superimposable to the usual dose in humans receiving 100–200 mg of doxycycline per day [44].

The toxicity of doxycycline hyclate has been found to be dose-dependent in 3 groups of 38-week-old male Wistar rats. The first group received no drug, the second 25 mg/kg twice per day, and the third group 50 mg/kg twice per day intragastrically [45]. The second group exhibited mild skeletal muscle injury compared with the intreated group as indicated with higher levels of creatine kinase and aspartate aminotransferase. In contrast, biopsies on the third group revealed significant skeletal muscle injury, cardiomyopathy and pulmonary lesions due to increased alveolar capillary pressure [45]. This study demonstrated that a daily dose of 100 mg/kg (tenfold higher than the standard therapeutic dose) could be poisoning, in contrast with 50 mg/kg per day (human equivalent dose) which is a very well tolerated dose in animal models [45].

## Discussion

Osteosarcoma aggression and metastases are strictly associated with the ability of tumour cells to secrete MMPs [3]. MMPs are secreted from normal tissues like smooth muscles cells and endothelial cells, supporting their ability to survive, expand and migrate participating in normal processes [4]. However, their presence in pathological tumourigenic processes is well identified. The expression of MMPs plays a key role in tumour spreading as they can modify the surrounding micro-environment of osteosarcoma cells. The extracellular matrix and basic membrane degradation accompanied by new vessel formation under direct or indirect action of MMPs provide an ideal frame of osteosarcoma cells to survive, expand and transport to distant organs [3, 13]. The supporting neo-angiogenesis promoted by MMPs conserves the survival of local tumours and the formation of lung-micro metastasis [4, 38]. Additionally, distant osteosarcoma metastatic lesions continue to secrete MMPs in order to adhere in normal tissues and form mature secondary tumours [21]. The construction of a supportive surrounding environment occurs with the formation of a new network of blood vessels.

To our knowledge, doxycycline has anti-MMP behaviour. Due to this function, it could be used not only as an antibiotic drug, but also as an adjuvant and neo-adjuvant chemotherapeutic agent. Doxycycline, as an MMP-inhibitor, blocks the potential growth and invasiveness of malignant cells as well as the formation of neoplastic new blood vessels and promises improvement in total survival rates in patients suffering from osteosarcoma [5, 21]. Doxycycline administration in the early stages of osteosarcoma expansion could diminish the mortality and expand the life expectancy of patients [28]. Due to the fundamental role of MMPs in highly metastatic malignant osteosarcoma cells, doxycycline showed a promising anti-cancer action by inhibiting MMPs. The tumor suppressing effect of doxycycline against osteosarcoma cells has been confirmed only by in vitro laboratory trials, since now. Published data from in vivo experimental trials in mice, have supported the anti-tumor effect of doxycycline in hepatocellular carcinoma, oral squamous-cell carcinoma, prostate cancer, duodenal adenocarcinoma and glioblastoma multiforme. Unfortunately, in vivo laboratory trials exploring the anti-metastatic action of doxycycline in animal models with primary osteosarcoma have not been conducted yet. Although there is strong evidence for the significant role of MMPs in cancer metastasis, the evidence for doxycycline as a chemotherapeutic agent in osteosarcoma remains weak. The lack of evidence consists a limitation for our research.

## Conclusion

Searching for published articles on pre-clinical experimental trials for the anti-metastatic effect of doxycycline as an MMP-inhibitor, we concluded that literature is lacking. We found only three pre-clinical in vitro trials, interpreting the tumour-suppressing effect of doxycycline via MMP-inhibition. Surprisingly, we found no available publications on this topic during the period from 1999 to 2013. MMPs play a key role in tumor metastasis, and doxycycline found to act as a significant anti-MMP factor. In our opinion, further osteosarcoma preclinical trials must be designed, especially high quality in vivo experimental studies to evaluate the usage and safety of doxycycline as an anti-neoplastic agent.

## Data Availability

All data generated for or analyzed in this study are included in this published article. We made references from published articles in PubMed and Google Scholar and all data supporting our findings can be found there. All figures included in this article were designed by its authors and they are attached in additional information files.

## Abbreviations

MMP: Metalloproteinase
VEGF: Vascular endothelial growth factor
ECM: Extracellular matrix
ALP: Alkaline phosphatase
DOX: Doxycycline
BFGF: Fibroblast growth factor
TGFβ: Tumor growth factor
